# Low level laser therapy (Photobiomodulation therapy) for breast cancer-related lymphedema: a systematic review

**DOI:** 10.1186/s12885-017-3852-x

**Published:** 2017-12-07

**Authors:** G. David Baxter, Lizhou Liu, Simone Petrich, Angela Spontelli Gisselman, Cathy Chapple, Juanita J. Anders, Steve Tumilty

**Affiliations:** 1 Centre for Health, Activity and Rehabilitation Research, School of Physiotherapy, University of Otago, Dunedin, New Zealand; 2 Department of Surgical Sciences, Southern District Health Board, Dunedin, New Zealand; 3 Department of Anatomy, Physiology and Genetics, Uniformed Services University of the Health Sciences, Maryland, MD USA

**Keywords:** Low level laser therapy, Photobiomodulation, Breast cancer related lymphedema, Systematic review

## Abstract

**Background:**

Breast cancer related lymphedema (BCRL) is a prevalent complication secondary to cancer treatments which significantly impacts the physical and psychological health of breast cancer survivors. Previous research shows increasing use of low level laser therapy (LLLT), now commonly referred to as photobiomodulation (PBM) therapy, for BCRL. This systematic review evaluated the effectiveness of LLLT (PBM) in the management of BCRL.

**Methods:**

Clinical trials were searched in PubMed, AMED, Web of Science, and China National Knowledge Infrastructure up to November 2016. Two reviewers independently assessed the methodological quality and adequacy of LLLT (PBM) in these clinical trials. Primary outcome measures were limb circumference/volume, and secondary outcomes included pain intensity and range of motion. Because data were clinically heterogeneous, best evidence synthesis was performed.

**Results:**

Eleven clinical trials were identified, of which seven randomized controlled trials (RCTs) were chosen for analysis. Overall, the methodological quality of included RCTs was high, whereas the reporting of treatment parameters was poor. Results indicated that there is strong evidence (three high quality trials) showing LLLT (PBM) was more effective than sham treatment for limb circumference/volume reduction at a short-term follow-up. There is moderate evidence (one high quality trial) indicating that LLLT (PBM) was more effective than sham laser for short-term pain relief, and limited evidence (one low quality trial) that LLLT (PBM) was more effective than no treatment for decreasing limb swelling at short-term follow-up.

**Conclusions:**

Based upon the current systematic review, LLLT (PBM) may be considered an effective treatment approach for women with BCRL. Due to the limited numbers of published trials available, there is a clear need for well-designed high-quality trials in this area. The optimal treatment parameters for clinical application have yet to be elucidated.

**Electronic supplementary material:**

The online version of this article (10.1186/s12885-017-3852-x) contains supplementary material, which is available to authorized users.

## Background

With improvements in early detection, diagnosis, and treatment of breast cancer, as well as an increase in breast cancer incidence, the number of breast cancer survivors is growing [1]. It is estimated that nearly 82% of women survive at least 10 years after diagnosis in developed countries (e.g. Europe, United States, and Japan) [1]. In New Zealand, the 10-year survival rate is estimated to be 92% with regular mammogram detection [2].

While this is encouraging, a considerable number of breast cancer survivors suffer from secondary lymphedema due to cancer related treatments (surgery and/or radiation therapy). Despite efforts to reduce lymphedema rates with new surgical techniques like the sentinel node biopsy technique replacing the axillary dissection as a standard for clinically node negative patients, breast cancer related lymphedema (BCRL) remains a relevant concern. A recent systematic review estimated that more than one out of five women who survive breast cancer are affected by BCRL [3]. This is in concordance with New Zealand specific data; it was estimated that the incidence of BCRL in New Zealand is 23.3% [4]. BCRL has a significant impact on breast cancer survivors, including declined physical function and increased disability, which negatively affects quality of life [5–8]. While the mainstay of BCRL management approaches include compression garments, manual lymphatic drainage, and remedial exercises [5, 9, 10], these interventions are usually time-consuming and poorly adherent (or unacceptable). There is a clear need for interventions to target the symptoms of BCRL and improve the wellbeing of these survivors.

**Fig. 1:**
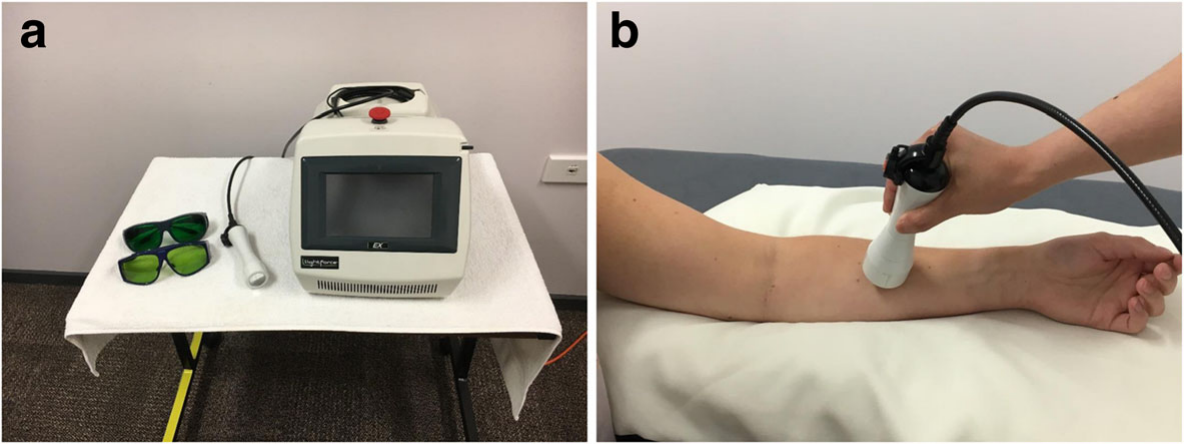
Examples of the technique of LLLT (PBM). **a** A device of LLLT (PBM). **b** Applying the LLLT (PBM) treatment head over a forearm region. Abbreviations: LLLT, low level laser therapy; PBM, photobiomodulation

Over the past two decades, low level laser therapy (LLLT) or photobiomodulation (PBM) has been promoted and researched for the management of BCRL. LLLT (PBM) is a non-invasive form of phototherapy that utilizes wavelengths of light between 650 and 1000 nm to deliver low irradiance and doses to the target tissue. It has been used to reduce inflammation, promote lymph vessel regeneration, improve lymphatic motility, and prevent tissue fibrosis [11–14]. It has been reported to be a safe technique [15]. Figure 1 illustrates an example of this technology.

Information on the basic mechanisms of LLLT (PBM) and a range of cellular effects have been demonstrated using a variety of cell types (fibroblasts; lymphocytes; osteoblasts; stem cells; smooth muscle cells) and in vitro [16–24]. These effects are the result of primary reactions involving absorption of specific wavelengths of light by components of the mitochondrial respiratory chain such as cytochromes, cytochrome oxidase, and flavin dehydrogenases; these result in changes in reduction–oxidation reaction (REDOX) status of cytoplasm and mitochondria, which in turn leads to increased levels of adenosine triphosphate (ATP) [25].

These primary reactions stimulate a cascade of secondary reactions at cellular level involving intracellular signalling and leading to stimulation of cytokine reactions, and nitric oxide (NO) production [17, 26]; release of growth factors [27–29]; up-regulation of ATP [30, 31], and increased metabolism, changes in REDOX signalling, increased reactive oxygen species (ROS) and therefore cell proliferation [30–32].In addition, stimulation of lymphatic vessels [33], and on lymphocytes [34] have been reported, as well as increases in local fluid circulation [35].

Previous literature reviews indicated promising effects of LLLT (PBM) for women with BCRL [15, 36, 37]. However, results were not robust due to a lack of formal synthesis methodology [15, 36], and the single meta-analysis did not perform subgroup analysis [37]. In order to address these issues, we aimed to conduct an updated systematic review of all available evidence from published clinical trials, including evidence from Chinese trials (with help of a Chinese collaborator), on the effectiveness of LLLT (PBM) for adult women with BCRL. Additionally, an assessment of treatment adequacy was carried out to examine the accuracy and clinical appropriateness of the treatment regimen of LLLT (PBM) in this area.

## Methods

### Protocol and registration

The review protocol was not registered.

### Search strategy

A comprehensive computer-aided literature search was undertaken in three English databases (PubMed, AMED, and Web of Science) and a Chinese database (CNKI) that includes grey literature (e.g. theses, conference proceedings), from their inception until November 2016. Search terms used were (cold laser OR laser OR laser light OR low-energy laser OR low-intensity laser OR low-level laser OR laser therapy OR photobiomodulation) AND (lymphedema OR lymphoedema OR swelling OR edema OR oedema) AND (breast cancer) with slight modifications for individual searches in each database (see Additional file 1 for search strategy). Additional articles were sought by manual screening of reference lists of all retrieved papers. Professionals working in the field were contacted to identify potential articles. Publication status was not restricted. No language restrictions were applied, provided there was an abstract available in English, as translation services were available.

### Inclusion criteria

Studies were considered eligible for inclusion if they satisfied the following criteria:

1. study design: clinical trials (e.g. randomized controlled trials (RCTs) and observational studies);
2. population: adult women who were diagnosed with BCRL;
3. intervention: LLLT/PBM therapy;
4. control (if applicable): there was no restriction regarding the control group, including no treatment or waiting list, sham therapy, and conventional therapy as any active treatment other than LLLT (PBM);
5. outcomes: clinically related outcome variables such as limb circumference/volume, pain intensity, range of motion, tissue resistance, tissue fluid, and subjective symptom.

### Exclusion criteria

Studies that included patients with primary lymphedema or lymphedema secondary to pathologic entities other than breast cancer related treatment were excluded. Reviews, guidelines, surveys, commentaries, editorials, and letters were excluded.

### Study selection

Two independent reviewers searched for potential articles by initially scanning the titles and abstracts to determine eligibility. Full papers were then reviewed for final inclusion. Differences between the reviewers were settled by discussion, and a third reviewer was consulted if differences persisted. Reviewers were not blinded to authors, institutions, publication journals, or study results.

### Data extraction

Data were extracted independently by the two reviewers using two standardized spreadsheets (one for RCTs and one for observational studies) designed to record author(s) and year of publication, study population, intervention, control comparison (if applicable), co-intervention, outcome measures, measurement time-points, conclusions and funding sources. Consensus was reached by discussion. Authors of original studies were contacted if further information was needed.

### Assessment of methodological quality

Methodological quality of included studies with RCT design was independently assessed by two reviewers using the physiotherapy evidence databases (PEDro) scale [38]. There was no blinding of study identification in this process. Before the assessment started, each item in the scale was intensively discussed to achieve consistency in the following procedure. Agreement level between the two reviewers was measured by the kappa statistic (kappa index less than 0.4 indicated poor agreement, 0.4 to 0.75 fair agreement, and over 0.75 excellent agreement) [39]. Again, consensus was reached through discussion. If a disagreement persisted, an independent decision was obtained from another collaborator. Since there are no accepted cutoff scores for the PEDro scale, a study was considered as high quality if the total score was 5 or higher [15, 36, 40]. The classification of quality was used to grade the strength of the evidence in data synthesis.

### Results synthesis

Primary analysis was based solely on the results from RCTs. The control groups, outcome measures, and the time points of follow-ups, were grouped according to the following criteria as a priori:

1. control comparisons: sham therapy which was physiologically inert; no treatment or waiting list; conventional therapy including compression bandages or garment, pneumatic compression pump, manual lymphatic drainage, complex decongestive therapy, and limb exercise;
2. outcome measures: primary outcome: limb circumference/volume; secondary outcome: pain intensity and range of motion;
3. time points: at discharge: immediately after end of all treatment sessions; short-term follow-up: <6 months after treatment; long-term follow-up: ≥ 6 months after treatment [41].

Outcomes of subgroup comparisons were summarized and evaluated. Meta-analysis was not performed due to the clinical heterogeneity and a limited number of included studies. Best evidence synthesis was conducted to generate final conclusions, taking into account the methodological quality, results of original studies, and numbers of RCTs that reported consistent findings (principal summary measures as effectiveness or non-effectiveness) [42]:

1. Strong—consistent findings (more than 75% of RCTs report the same findings) among multiple high quality RCTs;
2. Moderate—consistent findings among multiple low quality RCTs and/or one high quality RCT;
3. Limited—one low quality RCT;
4. Conflicting—inconsistent findings among multiple RCTs;
5. No evidence from trials—no RCTs.

### Assessment of treatment adequacy

LLLT (PBM) dosage parameters (e.g. wavelength, output power, power density (irradiance), energy density, and treatment area) of included studies (RCTs and observational studies) were used to judge the adequacy of treatment. Those parameters were compared to the World Association for Laser Therapy (WALT) guideline (https://waltza.co.za/documentation-links/recommendations/) [43]. Two reviewers who had extensive experience with research on laser applications independently assessed the adequacy and clinical appropriateness of the treatment dose, and resolved disagreement by discussion.

## Results

### Study selection

**Fig. 2:**
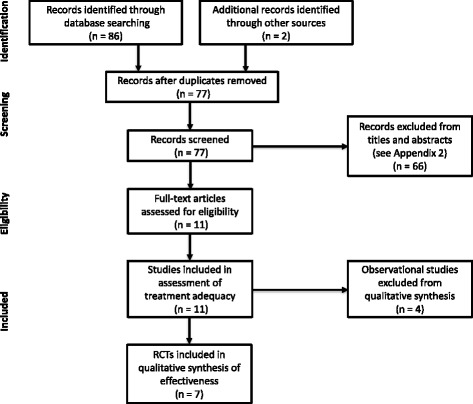
Flow diagram of literature search

In total, 88 studies were identified through electronic and hand searches. After excluding duplicates and those which did not meet the inclusion criteria, 11 studies were finally included (see Additional file 2 for excluded articles). An observational trial conducted by Piller and Thelander was regarded as two studies in this review due to different follow-ups (preliminary results (1995) [44] and main findings (1998) [45]). LLLT (PBM) treatment adequacy was assessed by these eleven studies. Data on the seven RCTs of the 11 studies were included in the effectiveness analysis; the remaining four studies were excluded as observational studies (Fig. 2).

### Study characteristics

Tables 1 and 2 present the main characteristics of the included RCTs and observational studies, respectively. All studies were published in English, and reported beneficial effects from LLLT (PBM). In the seven included RCTs, sample size ranged from 11 to 53. All trials measured limb circumference/volume, three (42.9%) measured pain intensity and range of motion. LLLT (PBM) was compared to sham laser therapy [46–48], conventional treatments including manual lymphatic drainage [49], pneumatic compression therapy [50] and compression bandage [51], and a waiting list control [52]. Follow-up lengths varied widely amongst the RCTs. Two trials ended immediately after the treatment regimen [49, 51], two trials followed patients for 1 month [48, 52], and another two trial assessed patient outcomes up to 2 [47] and 3 months [46], respectively. One RCT extended assessment time to 12 months [50], which was considered as long-term follow-up.

**Table 1:** Characteristics of 7 RCTs regarding LLLT (PBM) for BCRL

Authors (Year)	Participants	Interventions (No. Participants)	Co-intervention	Outcome Measures	Measured Time Points	Conclusions	Comments	Funding Resources
Ridner et al. (2013) [49]	46 women, unilateral BCRL	(1) LLLT (*n* = 15) (2) Manual lymphatic drainage (*n* = 16) (3) LLLT + manual lymphatic drainage (*n* = 15)	Compression bandaging after each Tx	I: Limb circumference	i: Baseline ii: Daily and weekly in Tx iii: Post-Tx	LLLT with bandaging may offer a time saving therapeutic option that provides similar results as those with conventional manual lymphatic drainage.	Small sample size; unaffected limb not assessed	ONS Foundation, National Center for Research Resources, the National Institutes of Health
Omar et al. (2011) [48]	50 women, unilateral BCRL	(1) LLLT (*n* = 25) (2) Sham laser (*n* = 25)	1) Limb exercise 2) Skin care instructions 3) Pressure garment	I: Limb circumference II: Range of motion	i: Baseline ii: 4 wk. iii: 8 wk. iv: 12 wk. v: 16 wk	LLLT was found to be effective in reducing the limb volume, increase shoulder mobility, and hand grip strength in approximately 93% of patients with post-mastectomy lymphedema.	Not with intention-to-treat analysis	NR
Lau and Cheing (2009) [52]	21 women, unilateral BCRL	(1) LLLT (*n* = 11) (2) Waiting list (*n* = 10)	Education	I: Limb volume	i: Baseline ii: Post-Tx iii: 4 wks post-Tx	LLLT was effective in the management of post-mastectomy lymphedema, and the effects were maintained to the 4wk follow-up.	Small sample size; assessor not blinded	NR
Kozanoglu et al. (2009) [50]	47 women, unilateral BCRL	(1) LLLT (*n* = 23) (2) Pneumatic compression therapy (*n* = 24)	1) Limb exercises 2) Hygiene 3) Skin care	I: Limb circumference II: Pain	i: Baseline ii: Post-Tx iii: 3 mo iv: 6 mo v: 12 mo	Both Tx modalities have positive effects in the treatment of post-mastectomy lymphedema, it seems that LLLT has better results at long term.	Small sample size; not with intention-to-treat analysis	NR
Maiya et al. (2008) [51]	20 women, unilateral BCRL	(1) LLLT (*n* = 10) (2) Compression bandage (*n* = 10)	Upper extremity exercise program	I: Limb circumference II: Pain	i: Baseline ii: Post-Tx	LLLT significantly reduces post-mastectomy lymphedema and pain compared to conventional group.	Lacked demographics; small sample size; lacked intragroup differences	NR
Kaviani et al. (2006) [47]	11 women, unilateral BCRL	(1) LLLT (*n* = 6) (2) Sham laser (*n* = 5)	NR	I: Limb circumference II: Pain III: Range of motion	i: Baseline ii: 3 wk. iii: 9 wk. iv: 12 wk. v: 18 wk. vi: 22 wk	LLLT may be effective in reducing arm circumference and pain, and in increasing the desire to continue Tx in patients with post-mastectomy lymphedema.	Very small sample size; not with intention-to-treat analysis	NR
Carati et al. (2003) [46]	61 women, unilateral BCRL	(1) LLLT (*n* = 33) (2) Sham laser (*n* = 28)	NR	I: Limb volume II: Range of motion	i: Baseline ii: Pre-Txs in C1 iii: End of C1 iv: Start of C2 v: End of C2 vi: 1 mo after C2 vii: 3 mo after C2	Two cycles of LLLT were found to be effective in reducing the volume of the affected arm, extracellular fluid, and tissue hardness in approximately 33% of patients with post-mastectomy lymphedema at 3 months after Tx.	Two-component crossover study, only 1st phase was included for analysis	AUSIndustry grant to RIAN Corp & Flinders University

*BCRL* breast cancer-related lymphedema, *C* cycle, *LLLT* low level laser therapy, *mo* months, *NR* not reported, *Tx* treatment, *wk.* weeks

**Table 2:** Characteristics of 4 observational studies regarding LLLT (PBM) for BCRL

Authors (Year)	Participants	Interventions (No. Participants)	Co-intervention	Outcome Measures	Measured Time Points	Conclusions	Comments	Funding Sources
Mayrovitz and Davey (2011) [54]	38 women, unilateral BCRL; 38 subjects (19 M/19 F), secondary leg lymphedema	(1) LLLT (*n* = 76) (2) Sham laser (*n* = 17 secondary ley lymphedema)	Manual lymphatic drainage: following (1) and (2)	Limb circumference	i: Baseline ii: Post-Tx iii: Post a manual lymphatic drainage	LLLT would reduce the skin water and tissue indentation resistance in patients with arm or leg lymphedema.	Observational study in Phase 1- LLLT (*n* = 76), sham laser in Phase 2- secondary leg lymphedema only	NR
Dirican et al. (2011) [55]	17 women, unilateral BCRL limited responsive to current therapy	LLLT (*n* = 17)	Conventional Tx	I: Limb circumference II: Pain III: Range of motion	i: Baseline ii: End of Cycle 1 iii: End of Cycle 2	Patients with BCRL received additional benefits from LLLT when used in conjunction with standard treatment. Two cycles were found to be superior.	Small sample size; statistical methods not clear	NR
Piller and Thelander (1995/ 1998) [44, 45]	10 women, unilateral BCRL	LLLT (*n* = 10)	Skin care instructions	I: Limb circumference II: Limb volume	i: Baseline, ii: Biweekly in Tx iii: 1 mo post-Tx iv: 3 mo post-Tx v: 6 mo post-Tx vi: 36 mo post-Tx*	LLLT, at least initially, improved most objective and subjective parameters of arm lymphedema.	Small sample size; lacked demographics; statistical significance unknown	Flinders 2000 & Flinders University

*36-months follow-up only applies to [45]

*BCRL* breast cancer-related lymphedema, *F* female, *LLLT* low level laser therapy, *M* male, *mo* months, *NR* not reported, *Tx* treatment

### Methodological quality assessment of RCTs

Results of the methodological quality assessment of the seven included RCTs are shown in Table 3. Inter-rater agreement was excellent in the independent assessment process (kappa index of 0.82), and consensus was reached after discussion. Among the seven RCTs, six (85.7%) were regarded to be of ‘high quality’. The major methodological quality issues with these RCTs were inappropriate concealed allocation (85.7%), lack of blinded trial assessor (85.7%), and lack of blinded therapists (71.4%).

**Table 3:** Quality assessment according to the PEDro scale (RCTs only)

Reference	1. Eligibility criteria	2. Random allocation	3. Concealed allocation	4. Baseline comparability	5. Blinded subjects	6. Blinded therapists	7. Blinded assessors	8. Adequate follow-up	9. Intention-to-treat analysis	10. Between-group comparisons	11. Point measures and variability	Total score (_/10)^¶^ (criteria 1 not included)
Ridner et al. 2013 [49]	Yes	Yes	No	Yes	No	No	No	Yes	Yes	Yes	Yes	**6**
Omar et al. 2011 [48]	Yes	Yes	No	Yes	Yes	No	Yes	Yes	No	Yes	Yes	**7**
Lau and Cheing 2009 [52]	Yes	Yes	No	Yes	No	No	No	No	No	Yes	Yes	4
Kozanoglu et al. 2009 [50]	Yes	No	Yes	Yes	No	No	No	Yes	No	Yes	Yes	**5**
Maiya et al. 2008 [51]	Yes	Yes	No	No	No	No	No	Yes	Yes	Yes	Yes	**5**
Kaviani et al. 2006 [47]	Yes	Yes	No	Yes	Yes	Yes	No	No	No	Yes	No	**5**
Carati et al. 2003* [46]	Yes	Yes	No	Yes	Yes	Yes	No	Yes	Yes	Yes	Yes	**8**
Sub-item total score (_/7)	7	6	1	6	3	2	1	5	3	7	6	

*Two-component crossover study, only the first phase was included for analysis

^**¶**^‘High quality’ studies (≥5) were represented in bold

### Effectiveness of LLLT (PBM)

Due to a limited number of eligible RCTs, only post-treatment and short-term follow-up comparisons (<6 months after randomization) could be assessed. Subgroup analyses were conducted as planned: in total, comparisons of three control groups for primary and secondary outcomes were made as below. Table 4 summarizes the results of individual studies.

**Table 4:** Summary of results of RCTs included in subgroup analysis

Studies	Limb circumference/volume		Pain intensity		Range of motion	
	Immediately after end of all sessions	Short-term follow-up (< 6 months)	Immediately after end of all sessions	Short-term follow-up (< 6 months)	Immediately after end of all sessions	Short-term follow-up (< 6 months)
LLLT (PBM) vs. sham laser						
Omar et al. 2011 [48]	+	+	NR	NR	+	NR
Kaviani et al. 2006 [47]	NR	+*	NR	+	NR	–
Carati et al. 2003 [46]	–	+*	NR	NR	–	–
LLLT (PBM) vs. conventional therapy						
Ridner et al. 2013 [49]	–	NR	NR	NR	NR	NR
Kozanoglu et al. 2009 [50]	+	–	–	–	NR	NR
Maiya et al. 2008 [51]	+	NR	+	NR	NR	NR
LLLT (PBM) vs. a waiting list control						
Lau and Cheing 2009 [52]	–	+	NR	NR	NR	NR

+: LLLT was more effective than the control group; −: LLLT was not more effective than the control group; *comparison at 1 month post treatment

*LLLT* low level laser therapy, *PBM* photobiomodulation, *NR* not reported

#### LLLT (PBM) versus sham laser (*n* = 3)

Three high quality studies [46–48] provided strong evidence that LLLT (PBM) was more effective than sham treatment for short-term (1 month post-treatment) total reduction in limb circumference. Two high quality studies [46, 48] provided conflicting evidence regarding the effects of LLLT (PBM) over sham laser on limb volume and shoulder mobility at the end of treatment. Two RCTs of high quality [46, 47] provided strong evidence suggesting similar effects from LLLT (PBM) and sham for range of movement in the affected limb in a short-term follow-up. There was moderate evidence (based upon a single high quality study [47]) supporting the effectiveness of LLLT (PBM) over sham laser for pain relief in a short-term follow-up (2 months post treatment).

#### LLLT (PBM) versus conventional therapy (*n* = 3)

Three high quality studies [49–51] provided conflicting evidence regarding differences between LLLT (PBM) and conventional therapy for short-term limb circumference reduction: two studies [50, 51] showed significant superior effects of LLLT (PBM) over compression (i.e. compression bandage and pneumatic compression) in limb girth at discharge; the other RCT [49] reported that LLLT (PBM) did not significantly differ in results from manual lymphatic drainage at the end of treatment. There was moderate evidence (one high quality RCT [50]) that LLLT (PBM) and pneumatic compression therapy were not significantly different at a 3-month follow-up. For secondary outcome measures, only pain intensity was compared; however, findings from two studies (high quality) produced contradictory conclusions. LLLT (PBM) was significantly superior to compression bandage for pain relief post treatment [51], whereas no significant differences were detected at treatment termination when compared with pneumatic compression [50]. There was moderate evidence (one high quality RCT [50]) showing an equivalent reductions in pain intensity level from LLLT (PBM) and pneumatic compression therapy at a short-term follow-up (3 months post treatment).

#### LLLT (PBM) versus a waiting list control (*n* = 1)

One RCT of low quality (*n* = 21) [52] found statistically significant effects of LLLT (PBM) in decreasing arm volume over no treatment at 4-weeks follow-up, yielding limited evidence in this comparison. However, no differences for such a comparison were found between these two groups immediately post-treatment (limited evidence).

### Application of LLLT (PBM)

Treatment parameters of LLLT (PBM) extracted from all 11 studies included in the review, and are displayed in Table 5. The standard of reporting of the laser parameters in the included studies was poor and did not follow WALT recommendations [53]. The most common wavelength used was 904 nm, reported in 6/11 studies [46, 48–50, 54, 55], three studies used a combination of two wavelengths [44, 45, 51], and one study failed to report the wavelength used [52]. When it was reported, the most common energy densities were 1.5 J/cm^2^ [46–48, 50] and 2.4 J/cm^2^ [44, 45, 51]. The common sites of application were the cubital fossa and the axillary region. Regimes typically delivered 3 treatments per week with variation in the duration of treatment from 4 weeks to 12 weeks. Three studies provided shorter treatment cycles with an 8 week stand-down between cycles [46, 47, 55].

**Table 5:** Treatment parameters of LLLT (PBM)

Study	Laser type/model	Treatment Area	Treatment Parameters	Laser parameters (output/power density/dose, when available)
Ridner et al. (2013) [49]	RianCorp LTU 904	Limb region	20–30 s/point; 20 min session	NR
Mayrovitz and Davey (2011) [54]	RianCorp LTU 904H	Limb region, 5 points	1 min/point; 5 min/session	5 mW output; 904 nm wavelength; in pulsed mode [Calculated: 0.3 J per point, 1.5 J total energy]
Dirican et al. (2011) [55]	RianCorp LTU-904	Axillary region, 17 points	1 min/point; two Tx cycles of 3 times/wk. for 3 weeks	0.3 J per point; 904 nm
Omar et al. (2011) [48]	Pagani IR27/4, GaAs	Antecubital fossa, 3 points; Axillary region, 7 points	2 min/point; 20 min/session, 3 times/wk. for 12 weeks	5 mW output; 904 nm; (maximum frequency of 2800 Hz, pulse duration of 50 ns); average dosage of 1.5 J/cm^2^
Lau and Cheing (2009) [52]	Comby 3 Terza Serie, Model D	Axillary region	Estimated Tx area of 144 cm^2^; 20 min/session, 3 times/wk. for 4 weeks	Three sources: 808 nm and ×2 905 nm, with outputs 24 mW–500 mW maximum. Combined emission mode with average dosage of 2 J/cm^2^
Kozanoglu et al. (2009) [50]	Electronica Pagani IR27/4, GaAs 904 nm	Antecubital fossa; Axillary region	20 min/session, 3 times/wk. for 4 weeks	904 nm wavelength in pulsed mode (frequency of 2800 Hz); dosage of 1.5 J/cm^2^
Maiya et al. (2008) [51]	He-Ne 632.8 nm laser device and Diode 850 nm laser	Axillary region	34 min/session, daily for 10 days	632.8 nm and 850 nm; dosage of 2.4 J/cm^2^
Kaviani et al. (2006) [47]	Mustang-024, GaAs diode laser device	Axillary region, 5 points	Two LLLT blocks (3 times/wk. for 3 weeks) with an 8-wk. interval (18 sessions in total)	10 W maximum output power, 890 nm wavelength in pulsed mode (frequency of 3000 Hz, pulse width of 130 ns, emission power of 4 mJ/s); dosage of 1.5 J/cm^2^
Carati et al. (2003) [46]	RianCorp LTU 904H	Axillary region, 17 points	1 min/point; 17 min/session; two LLLT blocks (3 times/wk. for 3 weeks) with an 8-wk. interval	5 mW average output; 904 nm wavelength in pulsed mode; dosage of 1.5 J/cm^2^
Piller and Thelander (1995/1998) [44, 45]	Space Mid M3-UP Helium Neon laser device	Axillary region; forearm; upper arm	30 min/session; 16 sessions (2 times/wk. for 6 weeks followed by 1 time/wk. for 4 weeks)	6.5 mw output power per course; 632.8 nm wavelength (Helium Neon laser); 14 mW average output power; 904 nm wavelength (semiconductor diode infrared lasers); treatment dosage ranged 2–4 J/cm^2^

*Corp* corporation, *min* minutes, *NR* not reported, *sec* seconds, *Tx* treatment, *wk.* week

## Discussion

The primary aim of this systematic review was to evaluate the effectiveness of LLLT (PBM) in the management of BCRL. Findings support the use of LLLT (PBM) for treating women with BCRL. Based upon the best evidence synthesis, the current review provided strong evidence (three high quality trials) favoring LLLT (PBM) over sham in terms of reduction in limb edema at short-term follow-up. For other comparisons, this review provided moderate evidence (one high quality trial) favoring LLLT (PBM) over sham for short-term pain relief, and limited evidence (one low quality trial) favoring LLLT (PBM) over no treatment for decreasing limb swelling at a short-term follow-up.

As a relatively novel therapeutic tool for the treatment of BCRL, LLLT (PBM) has gained increasing popularity since its approval by the United States Food and Drug Administration in 2007. Over the past two decades, seven RCTs [46–52] and four observational studies [44, 45, 54, 55] have been published in this area. Since RCTs are considered as the gold standard of contemporary medical research, the current systematic review generated conclusions about effectiveness of LLLT (PBM) based on the seven included RCTs. It is encouraging to note that the methodological quality of identified RCTs was ‘high’ in accordance with the PEDro scale (over 5/10); findings of this review were considered to be robust. Nevertheless, there was extensive study heterogeneity in treatment protocols, comparators, outcome measures, and follow-up periods. Due to a limited number of included studies, a head-to-head comparison to determine a superior LLLT (PBM) treatment regime was not possible. Future research into this area is suggested, which could provide evidence to guide development of an optimal LLLT (PBM) therapy regime for symptom management of BCRL.

This is the first systematic review applying best evidence synthesis to comprehensively evaluate the therapeutic value of LLLT (PBM) for BCRL. Findings from the review have strengthened conclusions of previous reviews [15, 36, 37], and confirmed the effectiveness of LLLT (PBM) in the treatment of BCRL. While two previous reviews [15, 36] showed favorable results of LLLT (PBM) in reduction of limb volume and tissue hardness, it was argued that these reviews lacked formal analysis methodology, thus reliability of the conclusions was unclear. Smoot et al. conducted a meta-analysis [37] to synthesize evidence from intervention studies, and concluded that there was moderate-strength evidence supporting the use of LLLT (PBM) in the management of BCRL. Although this review was rated as ‘moderate quality’ (6/11) according to the Assessment of Multiple Systematic Reviews (AMSTAR) criteria (a validated instrument for quality assessment of systematic reviews) [56], clinical appropriateness of pooling study results irrespective of control comparisons (lack of subgroup analysis) may limit the validity of the review conclusions.

Sham laser was typically set as a control arm in the included RCTs. Although the use of sham laser well satisfied the methodology requirement of double blinding to investigate the specific effects of LLLT (PBM), rationale for clinical utility of a novel treatment intervention (for instance, LLLT (PBM)) is best demonstrated against an accepted standard (best) therapy. This review found conflicting evidence regarding the effectiveness of LLLT (PBM) over conventional treatments, including manual lymphatic drainage, pneumatic compression therapy and compression bandage [49–51], on limb circumference and pain intensity. Another systematic review evaluating a series of conservative therapies has demonstrated that LLLT (PBM) yielded a similar percentage of volume reductions (approximately 11%) to compression garment or bandage [57]. Previous research suggested that wearing a compression garment alone results in a moderately significant reduction in BCRL [58]. Considering the intractable nature of BCRL, an integrative treatment package, in which LLLT (PBM) is used in addition to compression garment, may be a reasonable clinical option, and deserves further investigation through well-designed high quality RCTs.

Despite the clear statement by the WALT advocating standards of reporting of parameters when conducting studies involving laser therapy [53], there still seems to be inadequate information provided by authors of such studies. This is not uncommon and other systematic reviews have also highlighted these failures [59, 60]. Heterogeneity of the parameters used in the included studies and variable methods of application, along with differences in treatment regimes, all contribute to the difficulties of pooling information to make definitive statements regarding this use of LLLT (PBM) for BCRL. That being said, the normal genesis of treatment guidelines will result in many studies that show variation or contradictory results. Until patterns are recognized on a consistent basis across studies, the window of effective parameters cannot be identified. From all 11 studies included in this review, infrared wavelengths (808-905 nm) have been most commonly employed to date, and reported energy densities in the range of 1.5 J/cm^2^–2.4 J/cm^2^ have delivered positive outcomes. In comparison, effective energy densities for tendinopathy range from 1.8 J/cm^2^ to 19.2 J/cm^2^ depending upon the location of the tendon [59]. The reported frequency and duration of treatment is however too varied to make any strong statements, but a minimum of 4 weeks seems to be required.

The current review has adopted robust methodology to minimize the risk of bias. Firstly, it implemented most of the items listed in the AMSTAR checklist [56], therefore has a high methodological quality score (internal validity) of 9/11 (two points were missing because of the lack of a priori review protocol registry and an assessment of publication bias due to the qualitative analysis methodology). Secondly, in terms of the external validity, reporting of this review strictly adhered to the Preferred Reporting Items for Systematic Reviews and Meta-Analyses (PRISMA) guidelines [61] to ensure research replication. Thirdly, for data synthesis, subgroup analyses stratified by control comparisons and outcome measures were performed to address the influence of clinical (as well as statistical) heterogeneity. Fourthly, conclusions of the review were synthesized from seven RCTs with high methodology quality.

The primary limitation of this systematic review derived from the small number of included studies and lack of conclusions regarding the longer-term effects of LLLT (PBM) for BCRL management. Findings of this review suggest future well-designed fully powered RCTs are needed to inform the superiority of different LLLT (PBM) interventions, and determine an optimal treatment protocol for this therapy.

## Conclusions

Based upon the current systematic review, LLLT (PBM) in the management of BCRL is more effective for limb edema reduction than sham and no treatment at a short-term follow-up, and not more effective than other conventional treatments. Data suggest that LLLT (PBM) may be an effective treatment approach for women with BCRL. Due to the limited numbers of published trials available, there is a clear need for well-designed high-quality trials in this area. The optimal treatment parameters for clinical application have yet to be elucidated.

## Additional files

Additional file 1: Search strategy (PDF). This file presents the search strategy used in this systematic review in four databases (PubMed, AMED, Web of Science and CNKI). (PDF 193 kb)

Additional file 2: Excluded articles after duplicates removal (*n* = 66) (PDF). This file presents references of the 66 articles that were excluded after duplicates removed. (DOCX 23 kb)

## Abbreviations

AMSTAR: Assessment of Multiple Systematic Reviews
ATP: Adenosine triphosphate
BCRL: Breast cancer related lymphedema
LLLT: Low level laser therapy
NO: Nitric oxide
PBM: Photobiomodulation
PRISMA: Preferred Reporting Items for Systematic Reviews and Meta-Analyses
RCT: Randomized controlled trials
REDOX: Reduction–oxidation reaction
ROS: Reactive oxygen species
WALT: World Association for Laser Therapy
