# Supplementary material for: Low level laser therapy (Photobiomodulation therapy) for breast cancer-related lymphedema: a systematic review
Source: BMC Cancer. 2017 Dec 7;17:833. doi: 10.1186/s12885-017-3852-x (PMC5719569; doi:10.1186/s12885-017-3852-x)
Supplement: Supplementary file 1 — Search strategy (PDF). This file presents the search strategy used in this systematic review in four databases (PubMed, AMED, Web of Science and CNKI). (PDF 193 kb) [file 12885_2017_3852_MOESM1_ESM.pdf]

Additional file 1. Search strategy

1.1. PubMed (from inception to November 5th, 2016)

(1)	laser [MeSH Terms]	44,582
(2)	laser therapy [MeSH Terms]	53,144
(3)	laser light [MeSH Terms]	13,104
(4)	cold laser [MeSH Terms]	415
(5)	low-energy laser [MeSH Terms]	297
(6)	low-intensity laser [MeSH Terms]	306
(7)	low-level laser [MeSH Terms]	1,453
(8)	photobiomodulation [MeSH Terms]	0
(9)	or/(1)-(8)	92,858
(10)	lymphedema [MeSH Terms]	10,273
(11)	lymphoedema [MeSH Terms]	10,273
(12)	swelling [MeSH Terms]	38,980
(13)	edema [MeSH Terms]	38,980
(14)	oedema [MeSH Terms]	38,980
(15)	or/(10)-(14)	48,771
(16)	breast cancer [MeSH Terms]	243,481
(17)	(9) and (15) and (16)	14

1.2. AMED via Ovid Interface (from inception to November 5th, 2016)

(1)	laser.mp.	910
(2)	laser therapy. mp.	308
(3)	laser light. mp.	21
(4)	cold laser. mp.	4
(5)	low-energy laser. mp.	12
(6)	low-intensity laser. mp.	22
(7)	low-level laser. mp.	118

(8)	photobiomodulation. mp.	2
(9)	or/(1)-(8)	910
(10)	lymphedema. mp.	275
(11)	lymphoedema. mp.	116
(12)	swelling. mp.	632
(13)	edema. mp.	886
(14)	oedema. mp.	331
(15)	or/(10)-(14)	1,948
(16)	breast cancer. mp.	1,317
(17)	(9) and (15) and (16)	3

1.3. Web of Science (from inception to November 5th, 2016)

(1)	TS=(laser)	842,338
(2)	TS=(laser therapy)	31,185
(3)	TS=(laser light)	115,063
(4)	TS=(cold laser)	7,961
(5)	TS=(low-energy laser)	6,013
(6)	TS=(low-intensity laser)	2,349
(7)	TS=(low-level laser)	5,000
(8)	TS=(photobiomodulation)	616
(9)	or/(1)-(8)	842,489
(10)	TS=(lymphedema)	6,413
(11)	TS=(lymphoedema)	1,635
(12)	TS=(swelling)	117,461
(13)	TS=(edema)	87,437
(14)	TS=(oedema)	101,352
(15)	or/(10)-(14)	219,988
(16)	TS=(breast cancer)	434,706

(17) (9) and (15) and (16)	59

1.4. CNKI (from inception to November 5th, 2016), search conducted in Chinese

(1) 主题 (topic): 激光 (laser)	273,648
(2) 主题 (topic): 水肿 (edema)	121,080
(3) 主题 (topic): 乳腺癌 (breast cancer)	96,272
(4) (1) and (2) and (3)	10
