# Supplementary material for: Low level laser therapy (Photobiomodulation therapy) for breast cancer-related lymphedema: a systematic review
Source: BMC Cancer. 2017 Dec 7;17:833. doi: 10.1186/s12885-017-3852-x (PMC5719569; doi:10.1186/s12885-017-3852-x)
Supplement: Supplementary file 2 — Excluded articles after duplicates removal (n = 66) (PDF). This file presents references of the 66 articles that were excluded after duplicates removed. (DOCX 23 kb) [file 12885_2017_3852_MOESM2_ESM.docx]

**Additional file 2.** Excluded articles after duplicates removal (n= 66)

| **References** | **Reason for Exclusion** |
| --- | --- |
| Ackermann M, Wettstein R, Senaldi C, Kalbermatten DF, Konerding MA, Raffoul W, Erba P: **Impact of platelet rich plasma and adipose stem cells on lymphangiogenesis in a murine tail lymphedema model**. *Microvasc Res* 2015, **102**:78-85. | Not a clinical trial |
| Ancukiewicz M, Miller CL, Skolny MN, O'Toole J, Warren LE, Jammallo LS, Specht MC, Taghian AG: **Comparison of relative versus absolute arm size change as criteria for quantifying breast cancer-related lymphedema: the flaws in current studies and need for universal methodology**. *Breast Cancer Res Treat* 2012, **135**(1):145-152. | No intervention |
| Balci FL, DeGore L, Soran A: **Breast cancer-related lymphedema in elderly patients**. *Topics in Geriatric Rehabilitation* 2012, **28**(4):243-253. | Review |
| Basaran S, Kozanoglu E: **Breast cancer related lymphedema and conservative therapies**. *Turkish J Phys Med and Rehab* 2009, **55**(1):30-35. | Review |
| Brorson H: **Liposuction gives complete reduction of chronic large arm lymphedema after breast cancer**. *Acta Oncol* 2000, **39**(3):407-420. | Irrelevant intervention |
| Brorson H, Svensson H: **Skin blood flow of the lymphedematous arm before and after liposuction**. *Lymphology* 1997, **30**(4):165-172. | Irrelevant intervention |
| Brown MD: **Abstracts of the 41st Meeting of the British Microcirculation Society - April 5th-7th, 2004, University of Sheffield, Sheffield, UK**. *Microcirculation* 2004, **11**(6):527-555. | Conference proceeding |
| Cebicci MA, Sutbeyaz ST, Goksu SS, Hocaoglu S, Oguz A, Atilabey A: **Extracorporeal shock wave therapy for breast cancer-related lymphedema: A pilot study**. *Arch Phys Med Rehabil* 2016, **97**(9):1520-1525. | Irrelevant intervention |
| Chau N, Harris S: **Practices and opinions of physiotherapists treating patients with breast cancer-related lymphedema**. *Physiother Can* 2002, **54**(3):156-163. | Not a clinical trial |
| Cheng MH, Chen SC, Henry SL, Tan BK, Lin MCY, Huang J: **Vascularized groin lymph node flap transfer for postmastectomy upper limb lymphedema: Flap anatomy, recipient sites, and outcomes**. *Plast Reconstr Surg* 2013, **131**(6):1286-1298. | Irrelevant intervention |
| Cialdai F, Landini I, Capaccioli S, Nobili S, Mini E, Lulli M, Monici M: **In vitro study on the safety of near infrared laser therapy in its potential application as postmastectomy lymphedema treatment**. *J Photochem Photobiol B* 2015, **151**:285-296. | Not a clinical trial |
| Costa MM, Silva SB, Quinto ALP, Pasquinelli PFS, dos Santos VD, Santos GD, Veiga DF: **Phototherapy 660 nm for the prevention of radiodermatitis in breast cancer patients receiving radiation therapy: Study protocol for a randomized controlled trial**. *Trials* 2014, **15**. | Study procorol |
| Eisner A, O'Malley JP, Incognito LJ, Toomey MD, Samples JR: **Small optic cup sizes among women using tamoxifen: Assessment with scanning laser ophthalmoscopy**. *Curr Eye Res* 2006, **31**(4):367-379. | Irrelevant intervention |
| Eisner A, Toomey MD, Falardeau J, Samples JR, Vetto JT: **Differential effects of tamoxifen and anastrozole on optic cup size in breast cancer survivors**. *Breast Cancer Res Treat* 2007, **106**(2):161-170. | Irrelevant intervention |
| Fu MR, Ridner SH, Armer J: **Post-breast cancer lymphedema Part 2**. *Am J Nurs* 2009, **109**(8):34-41. | Review |
| Gottrup H, Andersen J, Arendt-Nielsen L, Jensen TS: **Psychophysical examination in patients with post-mastectomy pain**. *Pain* 2000, **87**(3):275-284. | Irrelevant intervention |
| Hayes SC, Johansson K, Stout NL, Prosnitz R, Armer JM, Gabram S, Schmitz KH: **Upper-body morbidity after breast cancer**. *Cancer* 2012, **118**:2237-2249. | Review |
| Jang DH, Song DH, Chang EJ, Jeon JY: **Anti-inflammatory and lymphangiogenetic effects of low-level laser therapy on lymphedema in an experimental mouse tail model**. *Lasers Med Sci* 2016, **31**(2):289-296. | Not a clinical trial |
| Karki A, Anttila H, Tasmuth T, Rautakorpi UM: **Lymphoedema therapy in breast cancer patients - A systematic review on effectiveness and a survey of current practices and costs in Finland**. *Acta Oncol* 2009, **48**(6):850-U871. | Review |
| Krok-Schoen JL, Oliveri JM, Kurta ML, Paskett ED: **Breast cancer-related lymphedema: Risk factors, prevention, diagnosis and treatment**. *Breast Cancer Manag* 2015, **4**(1):41-51. | Review |
| Kuz'mina EG, Degtiareva AA: **[Restoration of immunologic indices following reflexotherapy in the combination treatment of radiation-induced edema of the upper limbs]**. *Med Radiol (Mosk)* 1987, **32**(7):42-46. | Irrelevant intervention |
| Kuz'mina EG, Degtiareva AA, Doroshenko LN, Rogova NM, Zorina LN: **[Immunologic indices of the blood and interstitial fluid in the evaluation of the treatment of secondary edemas of the upper extremities]**. *Med Radiol (Mosk)* 1990, **35**(5):18-21. | Irrelevant population |
| Kuz'mina EG, Degtiareva AA, Zubova ND, Guseva LI, Klimanov ME: **[Effectiveness of various therapeutic schemes for patients with radiation edema of the extremities]**. *Med Radiol (Mosk)* 1987, **32**(3):18-22. | Irrelevant population |
| Lane KN, Dolan LB, Worsley D, McKenzie DC: **Upper extremity lymphatic function at rest and during exercise in breast cancer survivors with and without lymphedema compared with healthy controls**. *J Appl Physiol* 2007, **103**(3):917-925. | Irrelevant intervention |
| Lawenda BD, Mondry TE, Johnstone PAS: **Lymphedema: A Primer on the identification and management of a chronic condition in oncologic treatment**. *CA Cancer J Clin* 2009, **59**(1):8-24. | Review |
| Leal N, Carrara HHA, Vieira KF, Ferreira CHJ: **Physiotherapy treatments for bresat cancer-related lymphedema: A literature review**. *Rev Lat Am Enfermagem* 2009, **17**(5):730-736. | Review |
| Lin J, Jandial R, Nesbit A, Badie B, Chen MK: **Current and emerging treatments for brain metastases**. *Oncology-NY* 2015, **29**(4):250-257. | Irrelevant population |
| McKinnon JG, Wong V, Temple J, Galbraith C, Ferry P, Clynch GS, Clynch C: **Measurement of limb volume: Laser scanning versus volume displacement**. *J Surg Oncol* 2007, **96**(5):381-388. | No intervention |
| McNeely ML, Peddle CJ, Yurick JL, Dayes IS, Mackey JR: **Conservative and dietary interventions for cancer-related lymphedema: A systematic review and meta-analysis**. *Cancer* 2011, **117**(6):1136-1148. | Review |
| Merchant SJ, Chen SL: **Prevention and management of lymphedema after breast cancer treatment**. *Breast J* 2015, **21**(3):276-284. | Review |
| Mihara M, Hara H, Hayashi Y, Iida T, Araki J, Yamamoto T, Todokoro T, Narushima M, Murai N, Koshima I: **Upper-limb lymphedema treated aesthetically with lymphaticovenous anastomosis using indocyanine green lymphography and noncontact vein visualization**. *J Reconstr Microsurg* 2012, **28**(5):327-332. | Irrelevant intervention |
| Moseley AL, Carati CJ, Piller NB: **A systematic review of common conservative therapies for arm lymphoedema secondary to breast cancer treatment**. *Ann Oncol* 2007, **18**(4):639-646. | Review |
| e Lima MT, e Lima JG, de Andrade MF, Bergmann A: **Low-level laser therapy in secondary lymphedema after breast cancer: Systematic review**. *Lasers Med Sci* 2014, **29**(3):1289-1295. | Review |
| Nicoli F, Constantinides J, Ciudad P, Sapountzis S, Kiranantawat K, Lazzeri D, Lim SY, Nicoli M, Chen PY, Yeo MSW *et al*: **Free lymph node flap transfer and laser-assisted liposuction: A combined technique for the treatment of moderate upper limb lymphedema**. *Lasers Med Sci* 2015, **30**(4):1377-1385. | Irrelevant intervention |
| Okuno T, Kato S, Hatakeyama Y, Okajima J, Maruyama S, Sakamoto M, Mori S, Kodama T: **Photothermal therapy of tumors in lymph nodes using gold nanorods and near-infrared laser light**. *J Control Release* 2013, **172**(3):879-884. | Irrelevant population |
| Omar MT, Shaheen AA, Zafar H: **A systematic review of the effect of low-level laser therapy in the management of breast cancer-related lymphedema**. *Support Care Cancer* 2012, **20**(11):2977-2984. | Review |
| Oremus M, Dayes I, Walker K, Raina P: **Systematic review: Conservative treatments for secondary lymphedema**. *BMC Cancer* 2012, **12**. | Review |
| Paskett ED, Dean JA, Oliveri JM, Harrop JP: **Cancer-related lymphedema risk factors, diagnosis, treatment, and impact: A review**. *J Clin Oncol* 2012, **30**(30):3726-3733. | Review |
| Perbeck L, Celebioglu F, Svensson L, Danielsson R: **Lymph circulation in the breast after radiotherapy and breast conservation**. *Lymphology* 2006, **39**(1):33-40. | Irrelevant population |
| Poage E, Singer M, Armer J, Poundall M, Shellabarger J: **Demystifying lymphedema: Development of the lymphedema putting evidence into practice (R) card**. *Clin J Oncol Nurs* 2008, **12**(6):951-964. | Review |
| Powell K, Low P, McDonnell PA, Laakso EL, Ralph SJ: **The effect of laser irradiation on proliferation of human breast carcinoma, melanoma, and immortalized mammary epithelial cells**. *Photomed Laser Surg* 2010, **28**(1):115-123. | Not a clinical trial |
| Pula B, Olbromski M, Wojnar A, Gomulkiewicz A, Witkiewicz W, Ugorski M, Dziegiel P, Podhorska-Okolow M: **Impact of SOX18 expression in cancer cells and vessels on the outcome of invasive ductal breast carcinoma**. *Cell Oncol* 2013, **36**(6):469-483. | Not a clinical trial |
| Ridner SH, Montgomery LD, Hepworth JT, Stewart BR, Armer JM: **Comparison of upper limb volume measurement techniques and arm symptoms between healthy volunteers and individuals with known lymphedema**. *Lymphology* 2007, **40**(1):35-46. | No intervention |
| Rockson SG: **Diagnosis and management of lymphatic vascular disease**. *J Am Coll Cardiol* 2008, **52**(10):799-806. | Review |
| Rodrick JR, Poage E, Wanchai A, Stewart BR, Cormier JN, Armer JM: **Complementary, alternative, and other noncomplete decongestive therapy treatment methods in the management of lymphedema: A systematic search and review**. *Pm&R* 2014, **6**(3):250-274. | Review |
| Rogan S, Taeymans J, Luginbuehl H, Aebi M, Mahnig S, Gebruers N: **Therapy modalities to reduce lymphoedema in female breast cancer patients: A systematic review and meta-analysis**. *Breast Cancer Res Treat* 2016, **159**(1):1-14. | Review |
| Sanders JM, Butt L, Clark A, Williams J, Padgen M, Leung E, Keely P, Condeelis JS, Aguirre-Ghiso J, Castracane J: **A BioMEMS device for the study of mechanical properties of cells**. In: *Microfluidics, Biomems, and Medical Microsystems Xiii. Volume 9320*, edn. Edited by Gray BL, Becker H; 2015. | Not a clinical trial |
| Scardino MS, Swaim SF, Sartin EA, Hoffman CE, Oglivie GK, Hanson RA, Coolman SL, Davenport DJ: **The effects of omega-3 fatty acid diet enrichment on wound healing**. *Vet Dermatol* 1999, **10**(4):283-290. | Not a clinical trial |
| Smoot B, Chiavola-Larson L, Lee J, Manibusan H, Allen DD: **Effect of low-level laser therapy on pain and swelling in women with breast cancer-related lymphedema: A systematic review and meta-analysis**. *J Cancer Surviv* 2015, **9**(2):287-304. | Review |
| Stanton AWB, Levick JR, Mortimer PS: **Cutaneous vascular control in the arms of women with postmastectomy oedema**. *Exp Physiol* 1996, **81**(3):447-464. | Irrelevant intervention |
| Suarez Y, Gonzalez L, Cuadrado A, Berciano M, Lafarga M, Munoz A: **Kahalalide F, a new marine-derived compound, induces oncosis in human prostate and breast cancer cells**. *Mol Cancer Ther* 2003, **2**(9):863-872. | Irrelevant intervention |
| Tiwari P, Danish S, Madabhushi A: **Identifying MRI markers associated with early response following laser ablation for neurological disorders: Preliminary findings**. *PLoS ONE* 2014, **9**(12). | Irrelevant population |
| Trombetta C, Abundo P, Felici A, Ljoka C, Di Cori S, Rosato N, Foti C, Iop: **Computer aided measurement laser (CAML): Technique to quantify post-mastectomy lymphoedema**. In: *Young Researcher Meeting in Rome 2012. Volume 383*, edn.; 2012. | Conference proceeding |
| Yang GH, Shim JY: **The diagnosis and treatment of lymphedema**. *J Korean Med Assoc* 2013, **56**(12):1115-1122. | Review |
| Yanina IY, Tuchin VV, Navolokin NA, Matveeva OV, Bucharskaya AB, Maslyakova GN, Altshuler GB: **Fat tissue histological study at indocyanine green-mediated photothermal/photodynamic treatment of the skin in vivo**. *J Biomed Opt* 2012, **17**(5). | Irrelevant population |
| Zecha J, Raber-Durlacher JE, Nair RG, Epstein JB, Elad S, Hamblin MR, Barasch A, Migliorati CA, Milstein DMJ, Genot MT *et al*: **Low-level laser therapy/photobiomodulation in the management of side effects of chemoradiation therapy in head and neck cancer: Part 2: proposed applications and treatment protocols**. *Support Care Cancer* 2016, **24**(6):2793-2805. | Irrelevant population |
| Chen S, Cao J: **激光定时照射后口腔及颈部肿瘤血流动力学及影像动态分析**. *Journal of Clinical Rehabiliative Tissue Engineering Research* 2011, **11**(5):923-925. | Irrelevant population |
| Feng J: **糖基化聚氨基葡糖增强光动力治疗免疫效应的初步探讨 (Concomitant treatment with glycated chitosan improves immunological effect by photodynamic therapy)**. Guangzhou: Southern Medical University; 2015. | Irrelevant population |
| Feng Q: **PR-M对子宫平滑肌细胞线粒体膜电位作用的研究 (Impact of PR-M on mitochondrial membrane potential of uterine smooth muscle cells)**. Zhengzhou: Zhengzhou University; 2010. | Not a clinical trial |
| Li L: **99mTc-硫胶体乳腺癌前哨淋巴结探测系统性研究 (The systematic research of 99mTc-sulfur colloid in sentinel lymph node of breast cancer**. Jinan: University of Jinan; 2014. | Irrelevant population |
| Yang M: **高强度聚焦超声治疗鼠结直肠癌皮下移植瘤的实验研究 (A study of HIFU treatment in colorectal cancer subcutaneously transplanted murine model)**. Chongqing Chongqing Medical University; 2015. | Not a clinical trial |
| Zhang Z, Liu M, Cao S, Hu W: **超激光照射星状神经节预防乳腺癌术后上肢淋巴水肿40例**. *Shanxi Medical Journal* 2011, **40**(3):336-337. | Irrelevant intervention |
| Zhou X: **基于酵母微囊构建新型口服巨噬细胞靶向递送系统的研究 (Studies on novel macrophage targeted oral delivery systems based on the yeast shell)**. Chongqing Third Military Medical University; 2015. | Irrelevant population |
| Dong J: **磁共振导航高强度聚焦超声热消融羊肌肉组织的可行性研究 (An empirical study of mongolian gazelle muscle exposed to MR imaging guided high intensity focused ultrasound)**. Chongqing Chongqing Medical University; 2008. | Irrelevant population |
| Wu X: **紫苏子油纳米乳的研究 (The study on nanoemulsion of fructus perillae oil)**. Yangling: Northwest A&F University; 2008. | Irrelevant population |
| Sun J: **反义c-myc基因转染抑制后发性白内障的实验研究 (Adenovirus-mediated transfer of an antisense c-myc protooncogene into lens epithelial cells in vitro and in an experimental model of posterior capsule opacification)**. Qingdao: Qingdao University; 2004. | Irrelevant population |
